# Production of lipase and extracellular polymeric substances by the lipid-degrading bacterium *Burkholderia arboris* strain JYK2 in response to different substrates

**DOI:** 10.1007/s11274-025-04432-5

**Published:** 2025-06-12

**Authors:** Jiyu Huang, Mei-Fang Chien, Hernando P. Bacosa, Chihiro Inoue

**Affiliations:** 1 Graduate School of Environmental Studies, Tohoku University, Aoba Ku, Aoba 6- 6-20 Aramaki, Sendai, Miyagi 9808579 Japan; 2 Department of Environmental Science, School of Interdisciplinary Studies, Mindanao State University-Iligan Institute of Technology, Andres Bonifacio Avenue, Iligan, 9200 Philippines

**Keywords:** Lipid biodegradation, Lipase, Extracellular polymeric substance, *Burkholderia*

## Abstract

Characterization of lipid-degrading microorganisms and understanding their metabolite production mechanisms are essential for enhancing biodegradation efficiency of lipid-rich wastewater. In this study, we isolated *Burkholderia arboris* strain JYK2, which demonstrated lipid degradation at 46.29 mg/(l • h) with lipase activity reaching 59.92 U/ml when lipid was provided as the sole carbon source. Experimental results revealed that strain JYK2 secretes lipase in media containing lipids and fatty acids but not in glycerol-containing media, a phenomenon likely attributable to quorum sensing mechanisms. Furthermore, extracellular polymeric substances (EPS) were produced in the presence of lipids and fatty acids. Compositional analysis showed that EPS primarily consisted of proteins (approximately 50%) and polysaccharides (approximately 25%). This protein-rich characteristic conferred high hydrophobicity to the EPS, contributing to its lipase adsorption capacity as verified in this study. Additionally, biosurfactant activity was detected in the EPS produced by JYK2. These functions collectively enhance substrate utilization by microorganisms and promote lipid biodegradation. However, minimal EPS production was observed at low fatty acid concentrations, suggesting that the EPS production mechanism cannot be solely attributed to quorum sensing. This study provides insights into the interactions between lipid-degrading bacteria, lipase production, and EPS functionality, which are crucial for optimizing biological treatment of lipid-rich wastewater.

**Graphical Abstract:**

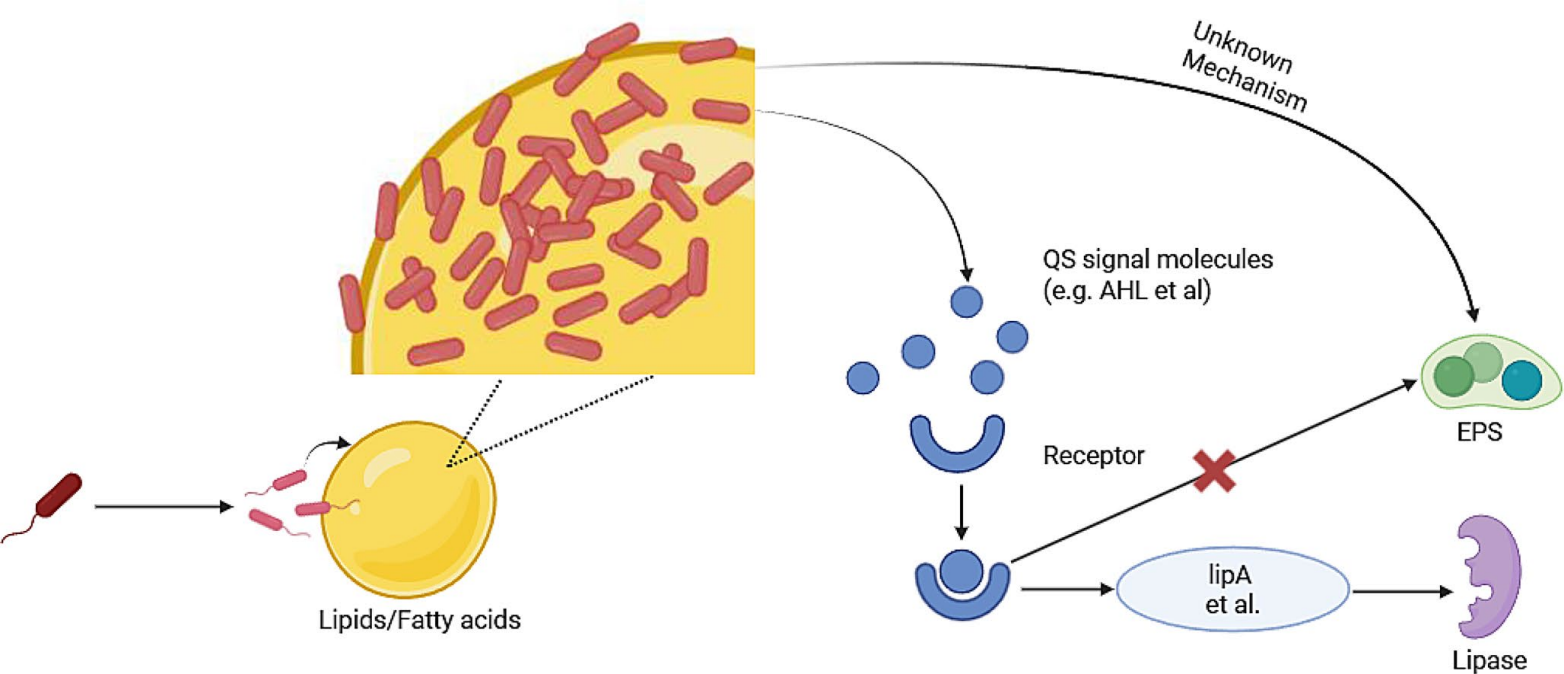

**Supplementary Information:**

The online version contains supplementary material available at 10.1007/s11274-025-04432-5.

## Introduction

With the change of lifestyle in recent years, the increasing demand for fat and oil has led to steady growth in the production and consumption of these products, resulting in discharge of significant amounts of lipid-rich wastewater. Biological treatments such as the activated sludge method are widely applied to manage this wastewater due to its low cost and high efficiency. Microorganisms play a crucial role in the removal of these substances from wastewater. To achieve better degradation of lipid-rich wastewater, understanding how microorganisms behave in the different processes in the system is crucial. Thus, characterizing these bacteria and elucidating their functions are necessary.

Lipid-degrading bacteria have been extensively studied, and various species have been found capable of producing lipases and degrading lipid content at high rates. These include *Rhodococcus sp*. (Ibrahim et al. 2020), *Enterobacter sp*. (Čipinytė et al. 2009), *Psuedomonas* sp. (Sharma et al. 2019), *Burkholderia sp*. (Ahmed et al. 2022; Chaiyaso et al. 2012), *Bacillus sp*. (Bhumibhamon et al. 2002; Tamilarasan and Kumar 2012), and *Serratia sp*. (Long et al. 2007). In addition to lipase production, some microorganisms facilitate lipid biodegradation by producing extracellular polymeric substances (EPS) to form biofilm, which not only protect bacteria from environmental stress but also increase the contact area between bacteria and lipids, thereby enhancing the bioavailability of lipids. Consequently, EPS has received increasing attention and is widely studied due to its crucial role in the biodegradation of various pollutants.

EPS is a general term for polymeric compounds produced outside of microorganism cells during their growth and are typically comprises polysaccharides, proteins, nucleic acids, and lipids. These substances are usually insoluble in water and can aggregate and adsorb to the exterior of cells, ultimately forming a micro-ecological system called a biofilm. The composition of EPS varies under different conditions and thus contributes to various functions; however, overall, EPS plays an important role in the interaction between cells and their surrounding environment. In terms of lipid degradation, many researchers have noted that EPS can not only emulsify aggregated lipid droplets through its biosurfactant component (Flemming et al., 2010; More et al. 2014; Soberón-Chávez et al. 2005), but also immobilize cells and lipids within the biofilm, facilitating substrates utilization and improving degradation efficiency (Ding et al. 2015).

Given the importance of both lipase and EPS in lipid biodegradation, elucidating the production mechanism of these substances is essential. Some researchers have suggested that quorum sensing (QS), a fundamental microbial communication system based on population density (Miller & Bassler. 2001), plays an important role in the production of these substances. QS regulates various metabolic pathways, including those for lipase, biosurfactant, and other metabolites. Zeng et al. (2024), Devescovi et al. (2007) and Bai & Vittal (2014) elucidated that QS regulates lipase production in various microorganisms belonging to *Burkholderia sp.* and *Pseudomonas sp.* Zhang et al. (2012) showed that QS can regulate the expression of rhamnolipid production genes *rhlA* and *rhlB.* However, the role of QS in EPS production remains debated due to its complexity. Ueda and Wood (2009) reviewed the production mechanism of several EPS components by *Pseudomonas aeruginosa*, revealing that while some exopolysaccharides are QS-regulated, most EPS components are controlled by other mechanisms. Thus, the production mechanism of EPS remains unclear.

Recent studies on EPS have primarily focused on microbial consortia in sludge systems, with an emphasis on the role of EPS in enhancing lipid biodegradation. For instance, Wang (2023) demonstrated that EPS produced during sludge treatment of lipid-rich wastewater functions as a biosurfactant, while Xu (2015) and Yang (2016) reported that EPS contributes to increased biogas production during the anaerobic co-digestion of sludge and lipids. However, these studies did not isolate and analyze EPS produced by individual bacterial strains, making it difficult to elucidate the specific mechanisms involved in EPS production and function. In contrast, some studies have investigated EPS produced by pure cultures. For example, Guibaud (2005), Zhao (2015), Subramanian (2010), and Bramhachari (2007) examined the physicochemical properties of EPS under controlled single-strain cultivation. Nevertheless, these studies commonly used glucose as the sole carbon source and did not explore how lipids or fatty acids influence EPS production. As a result, little is known about the EPS production mechanisms when lipid-degrading bacteria are cultured with lipid-based substrates. To address this gap, the present study aims to investigate the production and functional mechanisms of EPS and lipase under various carbon sources, using a lipid-degrading, EPS-producing bacterium, *Burkholderia arboris* JYK2, which was isolated from a fallow field.

## Materials and methods

### Sampling and pretreatment

The soil samples were collected from five locations in Japan in September 2018. Samples K, I and T were obtained from a farmland in Kesen-numa, Sendai and Tome, Miyagi prefecture. Samples F and A were collected from a forest in Iidate, Fukushima prefecture and Sendai, Miyagi prefecture. We collected soil samples from forest and farmland areas since these environments usually have high organic matter content which may support the growth of lipid-degrading microorganisms. The samples were stored at 4 °C before enrichment cultivation.

The lipids used in this study were the mixture of vegetable oils (canola oil and salad oil). They were purchased from the local market and mixed at 1:1. The fatty acids used in this experiment were separately oleic acid, linoleic acid, and palmitic acid mixed in 6: 3: 1, whose ratio is closer to the fatty acid composition of canola oil and sunflower oil. The mixed oil and fatty acid were stored at room temperature in the dark.

### Screening and identification of lipid fast-degrading bacteria

#### Screening of lipid fast-degrading consortia

The bacterial consortia for degrading lipids at a high rate were enriched in a 300 ml Erlenmeyer flask containing 100 ml of mineral salt medium (MSM) with 0.1 g/l of yeast extract and 1% (v/v) of the mixed vegetable oil. The MSM contained (NH_4_)_2_SO_4_ (5 g/l), K_2_HPO_4_ (2 g/l), NaH_2_PO_4_ (2 g/l), while pH was adjusted to 7.0. Before inoculation, the flask containing MSM and yeast extract was sterilized at 121℃ for 30 min in an autoclave. While the mixed oil was sterilized by irradiation of UV light (280 nm) for 30 min. Then, 1 g of soil sample was inoculated to the flask containing sterilized liquids and incubated at 30 °C, shaking at 150 rpm for 14 days. Subculture to next generation was done biweekly with 1% (v/v) inoculum. After 6 times of subculture, the lipid concentrations on the 7th day and 14th day were measured to screen the consortium capable of degrading mixed oil rapidly.

#### Identification of lipid-degrading bacteria

Isolation of lipid-degrading bacteria was done using LB agar plate (10 g/l tryptone, 5 g/l yeast extract, 10 g/l NaCl, 15 g/l agar). After spreading cell suspension on the plate and incubating at 30 °C for two days, single colonies with distinct morphology were selected and incubated in LB medium, taken after a continuous purification process. Their ability to degrade mixed oil and their lipase activity were further tested for screening purposes. The culture medium of qualified strains was mixed with 50% glycerol to a final concentration of 3% stored in -80 °C for further experiment.

The genomic DNA of isolated strains were extracted using the Wizard^®^ Genomic DNA Purification Kit, according to the manufacturer’s manual. The 16S rRNA gene was amplified by polymerase chain reaction (PCR) using primers 27F (5’-AGAGTTTGATCCTGGCTCAG-3’) and 1492R (5’- GGTTACCTTGTTACGACTT-3’). The PCR condition was as follows: denaturation at 95 °C for 2 min; 30 cycles of denaturation at 95 °C for 15 s, annealing at 55 °C for 20 s, and primer extension at 72 °C for 90 s; and a final extension at 72 °C for 7 min. The PCR products were cleaned up according to the protocol of NucleoSpin^®^ Gel and PCR Clean-up and then sent to Macrogen Japan Corp. (Tokyo, Japan) for sequencing. The sequencing results were treated by software Codoncode Aligner (version 5.1.4.6) and submitted to GenBank database and compared with sequence of most related microorganisms via BLAST.

### Degradation characteristics analysis experiments of strain JYK2

To examine further the characteristics of isolate strains, a batch of degradation experiments were conducted to evaluate their ability to degrade lipids and produce lipase. The best lipid degrader and lipase producer was found to be strain JYK2, and its ability to degrade lipids, fatty acid, or glycerol, producing lipase and EPS, was further studied. After being taken out of the refrigerator, the strains were first precultured in LB broth for 3 days, and then 1% of preculture medium inoculated in basic medium addition with different carbon sources for 12 days. Samples were regularly collected for the following analysis.

#### Analysis of substrates consumption

The substrates analysed in this study included mixed vegetable oil, referred to as lipids, mixed fatty acid, and glycerol. Residual mixed vegetable oil was extracted by n-hexane and weighed by the gravimetric method (Sihag and Pathak 2016). The detailed steps were as follows. First, n-hexane was added to the samples under slightly acidic conditions. Then, the mixed liquid was severely vortexed and then centrifuged at 1,400 × g in 10 min for layering. The hexane phase was transferred to a clean, pre-weighed test tube and heated to 80 °C to evaporate the hexane, allowing the lipid content to remain in the vessel. The following equation calculated the lipids degradation ratio:

$$\:Lipids\:degradation\:ratio=\:1-\:\frac{oil\:weight\:change}{total\:oil\:weight}\:\times\:100\%$$

Due to the fact that long-chain fatty acids have similar properties to oil, they were also measured by the gravimetric method and extracted by n-hexane. Thus, the fatty acids degradation ratio was calculated by a similar equation:

$$\:Fatty\:acids\:degradation\:ratio=\:1-\:\frac{FA\:weight\:change}{total\:FA\:weight}\:\times\:100\%$$

In contrast, glycerol can be easily dissolved in water and thus cannot be extracted by n-hexane; therefore, the amount of residual glycerol in the medium was roughly measured by total organic compounds (TOC) (Schumacher 2002). Considering that the remaining organic carbon in the culture medium mostly consists of soluble polysaccharides and proteins produced by cell secretion or lysis, these parts of organic compounds were deducted. The soluble polysaccharides and proteins were separately measured by the sulfuric acid-phenol colorimetric method and modified Lowry method, and the residual glycerol in the medium was calculated by the following equation:

$$\:Glycerol\:degradation\:ratio=\:1-\:\frac{TOC-{\left[PS\right]}_{c}-{\left[PN\right]}_{c}}{initial\:carbon\:mass\:\:in\:glycerol\:}\:\times\:100\%$$

[PS]_C_: polysaccharide converted to carbon mass.

[PN]_C_: protein converted to carbon mass.

#### Bacterial growth

The growth condition of bacteria was assayed using a combined method that incorporated turbidity measurement and direct microscopic count. Briefly, 200 µL of medium was added into an Eppendorf tube and centrifuged in 15,000 × g for 2 min; supernatant was taken out for EPS test later while PBS buffer solution was added into tubes to resuspend the cells. This step was repeated three times, and finally, the total volume of the cell PBS suspension was adjusted to 1000 µL. The optical density of the suspension in 600 nm (OD600) was measured regularly. Then, an OD-measured sample and its dilution were sequentially added into a hemocytometer, and a microscope was used to count the number of bacteria in specific squares of the grid. The total number of bacteria was calculated based on the volume of liquid in the chamber and the number of bacteria counted. Therefore, a standard curve of OD600 and bacteria number could be obtained, and growth conditions were finally represented by bacteria number in a specific volume calculated by this method.

#### EPS amount

Different from EPS from natural water bodies and wastewater treatment plants, EPS obtained in this study were produced from a single strain and cultured in a simple medium. Therefore, the quantification of EPS in the media was measured by combining turbidity measurement and standard method 2540D (Dramais, 2018) with modification, which was able to measure the suspended solids in water. Like the assessment method of bacteria growth, all the supernatant separated from step 2.3.2 were collected as an EPS source, and OD600 was measured to roughly represent the EPS amount. Then, an OD-measured sample and its dilution were prepared and sequentially passed pre-weighed 0.22 μm filters. Milli-Q water was then filtered three times as well to wash the filter. Afterward, the filters captured EPS were placed in a drying oven for 24 h. By this method, a standard curve of OD600 and EPS weight can be drawn, allowing EPS amount to be indicated by weight in a specific volume.

#### Lipase activity

Lipase activities of strains were measured by p-nitrophenol (p-NP) method with slight modification (Gaur et al. 2008; Kilcawley et al. 2002; Yoo et al. 2011). Briefly, 900 µl of solution A containing 1 mM CaCl_2_ and 0.1%(v/v) Triton X-100 in 0.1 M Tris/HCl buffer (pH 8.0), 80 µl of 10 mM p-nitrophenol palmitate (p-NPP) in acetonitrile solution and 20 µl of culture medium filtered by 0.22 μm filter were mixed and incubated at 30 °C for 30 min. Then, 10 µl of ZnSO_4_ solution added to terminate the reaction and the mixture immersed in ice for 5 min. After centrifugation of the mixture at 10,000 x g for 10 min, the absorbance of the supernatant measured at 405 nm using a microplate reader. Lipase activity was expressed in unit (U), where one unit will release 1 µM p-NP from p-NPP per minute in 1 ml of the assay solution.

### Composition and function assay of EPS

#### Determination of major component

The amount of polysaccharide and protein content in EPS was measured for the supernatant of the bacterial growth experiment using 20 ml/l of lipid as the substrate (Sect. 2.3.2). The detail of experiment was designed as follows (Zuriaga-Agustí et al. 2013): one cell-free suspension sample was divided into two assay tubes; one tube was treated with Triton-X-100 to depolymerize the EPS matrix into smaller components, while another was added equivalent Milli-Q water (control). Both tubes were filtered through 0.22 μm membrane filter and reserved filtrates to measure the polysaccharide and protein content. EPS in control tube cannot pass through the 0.22 μm filter, so that the filtrate should only contain soluble substances in the cell-free suspension. In comparison, the filtrate treated with Triton-X-100 should contain both depolymerized EPS components and soluble substances. Thus, the major components of EPS in the cell-free suspension can be quantified by comparing the difference between two tubes.

#### Localization of lipase in cell-free suspension

To determine whether lipase is immobilized to EPS or not, lipase activity of the solutions in the two assay tubes described in Sect. “Determination of major component” was measured. The method for measuring lipase activity is the same as that described in Sect. “Lipase activity”

#### Biosurfactant assays

The biosurfactant activity of EPS was verified by the oil displacement assay (ODA) method developed by Morikawa et al. (Morikawa et al. 1993). Briefly, 200 µl of vegetable oil dyed by Sudan III was put onto the surface of 20 ml of Milli-Q water in a petri dish. After a thin oil layer formed, 20 µl of EPS contained sample or control sample (Milli-Q water) was gently added to the top of the oil layer. The formation of a clear halo evaluated as a criterion of biosurfactant activity.

### Statistical analysis

For the experiments conducted in triplicate (*n* = 3), the data is presented as mean ± standard deviation (SD). Statistical significance was evaluated using Welch’s t-test and one-way ANOVA, where Welch’s t-test was applied when comparing the means between two groups, and one-way ANOVA was used for assessing statistical differences among three or more groups. Graphs were generated with error bars representing SD to indicate data variability.

## Results

### Enrichment of lipid-degrading consortia

The forest soil and field soil are generally believed to contain high organic and lipid content and thus likely harbors lipid-degrading microorganisms. We collected soil samples from five locations covered by fallen leaves or cultivated oil crops such as soybeans. After five generations of subculture, lipid degradation ratios of the enriched consortia were measured (Fig. S1). The lipid degradation rate of each bacterial consortium on day 7 and day 14 was significantly different from that of the control group (*n* = 3, *p* < 0.05), in which consortia enriched from Kesen-numa (K), Fukushima (F) and Tome (M) showed superior lipid degradations at day 7 compared to other consortia, reaching 70.78 ± 8.07%, 68.49 ± 12.92% and 62.79 ± 6.46% respectively. The corresponding 7-day lipid-degrading rate were 39.17 ± 4.47, 37.91 ± 6.26 and 34.75 ± 3.57 mg/(l • h) separately. In contrast, other consortia failed to achieve 50% of lipid degradation ratio. By day 14, the degradation ratio of K, F, and M remained stable at approximately 70%, reaching 67.35 ± 16.14%, 65.07 ± 10.33%, and 69.63 ± 9.69%, respectively, while other consortia remained below 60%. Based on these degradation rate and kinetics consortia K, F, and M were selected for further analysis.

### Isolation and characterization of lipid-degrading bacteria

Lipid-degrading strains were isolated using LB agar plates, with 3 strains isolated from each consortium based on morphological differences. The isolates were identified by 16 S rRNA sequence analysis and database comparison (Table S1). Strains with 16 S rRNA sequence identity above 98% were classified as the same species. Accordingly, strain JYK1, JYK2, JYK3, JYF3, and JYM2 were identified as *Burkholderia arboris*; JYF1 as *Methylobacterium radiotolerans*; JYF3 and JYM3 as *Klebsiella aerogenes*; and JYM1 as *Serratia marcescens.*

**Fig. 1:**
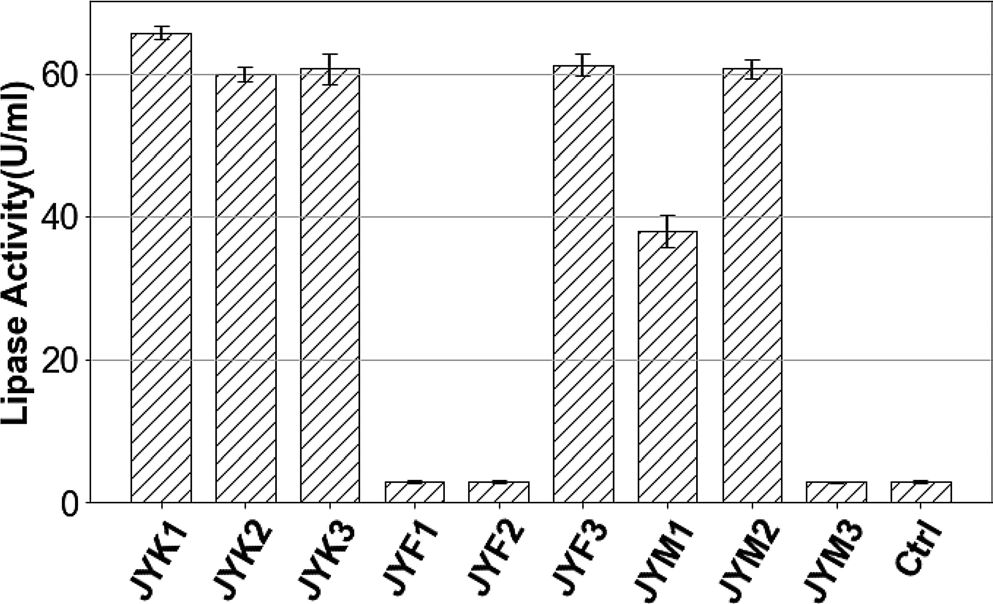
Lipase activity of isolated strains (Day6): Strains JYK1 ~ 3 were isolated from Kesennuma, JYF1 ~ 3 were isolated from Fukushima and JYM1 ~ 3 were isolated from Tome. Ctrl referred to a negative control group without bacteria inoculation in the medium

Lipase activity and oil degradation ability of these isolated strains were analyzed (Fig. 1 and Fig. S2, respectively). Among these lipase-producing strains, five *B. arboris* strains (JYK1, JYK2, JYK3, JYF3, JYM2) showed excellent lipase activity (59.91 ± 1.02 ~ 65.73 ± 1.01 U/ml). In comparison, lipase produced by JYM1 (*S. marcescens*) reached only 37.95 ± 2.32 U/ml. All six strains showed statistically significant differences compared with the control group (*n* = 3, *p* < 0.05). The lipid degradation analysis revealed consistent trends: lipase-producing strains exhibited significantly higher degradation ratios than the control group from day 4 to day 14 (*n *= 3, *p* < 0.05). Strain JYK2 achieved the highest degradation ratio of 81.05 ± 5.24% by day 14, while JYM1 degraded less oil, likely due to its lower lipase activity. The remaining strains degraded less than 40% of lipid, substantially lower than the lipase-producing strains. Table 1 summarized the lipase activity and lipid degradation ratio of each strain on day 6. Based on its superior lipase activity and lipid-degrading ability, strain JYK2 was selected for further analysis. This strain has been deposited at the Microbe Division, RIKEN BioResource Research Center (JCM 37745 *B. arboris*, accession number: PQ373032).

**Table 1:** Lipase activity and lipid degradation rate of isolated strains

Strain	Lipase activity (U/ml)	Lipid degradation rate (mg/(l • h))
JYK1	65.73	44.61
JYK2	59.92	46.29
JYK3	60.66	41.05
JYF1	2.90	10.55
JYF2	2.88	27.30
JYF3	61.22	44.02
JYM1	37.95	32.25
JYM2	60.66	41.47
JYM3	2.82	28.33

Lipid degrading and lipase producing microorganisms have been extensively studied over the past two decades, with various species capable of degrading lipids under diverse conditions. Table 2 compares the lipid-degrading ability and lipase activity of several bacteria cultured under similar conditions to this study (MSM with approximately 10 ml/l vegetable oil as substrate) with those of *B. arboris* strain JYK2.

**Table 2:** Comparison of studies on lipid-degrading bacteria and lipase-producing bacteria

Microorganisms	Lipase Activity (U/ml)	Lipid Degrading Rate (mg/ (l • h))	Culture Condition	Reference
*Enterobacter sp.*	0.45	61.9	MSM + Sunflower oil	(Čipinytė et al. 2009)
*Arthrobacter sp.*	0.41	66.6	MSM + Sunflower oil	(Čipinytė et al. 2009)
*Penicillium sp.*	47.61	Not Discussed	MSM + Waste cooking oil	(Kumar et al. 2012)
*Burkholderia sp.*	12.1	44.3	MSM + Palm oil	(Lo et al. 2012)
*Burkholderia arboris*	59.92–65.73	41.05–46.29	MSM + Vegetable Oil	This study

Čipinytė et al. (2009) reported that *Enterobacter* sp. and *Arthrobacter* sp. degraded sunflower oil in MSM at rates of 61.9 and 66.6 mg/(l • h), respectively, but with lipase activity of only 0.45 and 0.41 U/ml. Lo et al. (2012) found that *Burkholderia sp.* degraded lipids at a rate of 44.3 mg/(l • h) with lipase activity of 12.1 U/ml. Kumar et al. (2012) reported that *Penicillium sp.* cultured in waste cooking oil produced lipase with activity of 47.61 U/ml. Based on these comparisons, strain JYK2 demonstrates moderate lipid degradation ability but exceptional lipase activity compared to other bacteria.

### Lipase activity of strain JYK2 in EPS

**Fig. 2:**
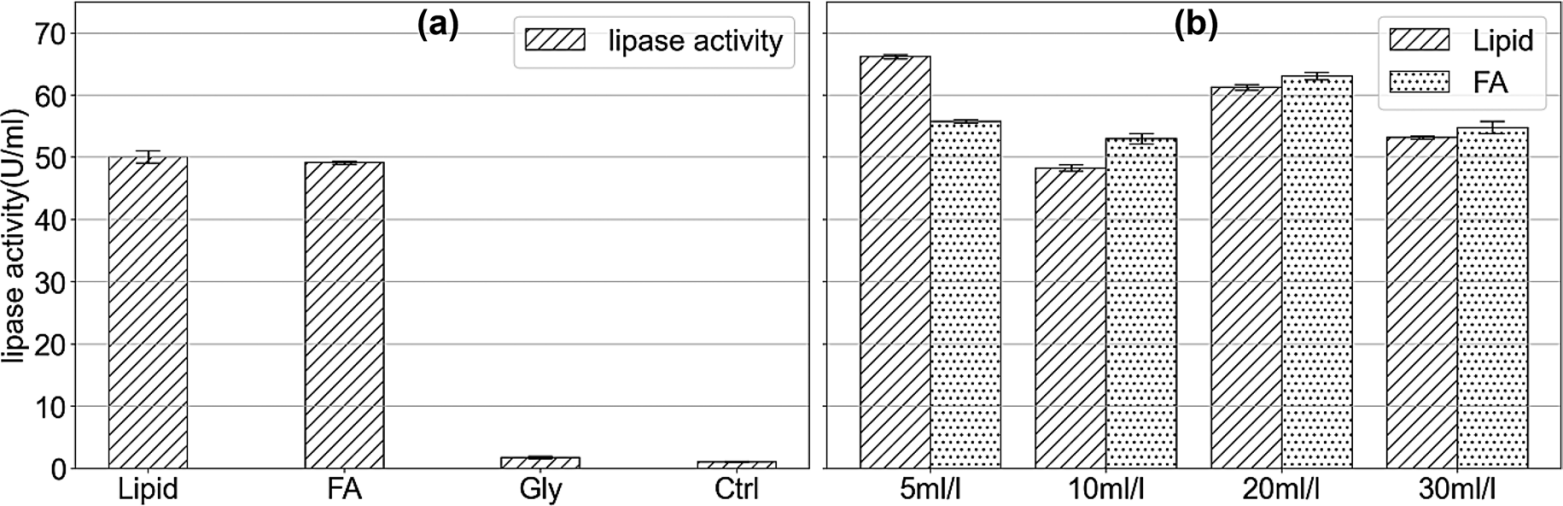
Lipase activity in different substrates (**a**) or different initial concentrations of lipid and fatty acid (**b**). FA- fatty acid, Gly-glycerol and Ctrl-Milli-Q water

Lipase plays a crucial role in lipid biodegradation by catalyzing the hydrolysis of triglyceride molecules (the main components of oils and fats) into free fatty acids and glycerol, which can then be absorbed and metabolized by bacteria. In this study, lipase activity in the culture supernatant was quantified by p-NP method. Figure 2(a) shows the lipase activity produced by strain JYK2 when cultured with different substrates. Significantly higher lipase activity (approximately 50 U/ml) was observed when the vegetable oil (lipid) and fatty acid were used as substrates compared to the control group (*n* = 3, *p* < 0.01). In contrast, minimal lipase was produced when glycerol served as the carbon source. For vegetable oil and fatty acid, lipase activity at different initial concentrations was also examined (Fig. 2(b)). Interestingly, lipase activity remained relatively constant despite increasing substrate concentrations.

### Biodegradation of different lipid-related substances by strain JYK2

#### Degradation ratio of lipids and their hydrolysis byproducts

Degradation experiments were conducted to determine the degradation kinetics of lipids and their hydrolysis byproducts (fatty acid and glycerol). Fig. S3 presents the degradation ratio of three substrates by strain JYK2 over 12 days at various initial concentrations. For lipids, degradation ratio increased over time at all initial oil concentrations, indicating active bacterial degradation. However, the degradation pattern was not linear; it increased rapidly during the first 4 days, followed by a steady increase until day 9, and then slightly decreased or plateaued. At initial concentration of 5 ml/l and 10 ml/l, the degradation ratio reached 79.36 ± 10.17% and 84.88 ± 7.18%, respectively, by day 12. In contrast, at higher concentrations (20 ml/l and 30 ml/l) the degradation ratio was significantly lower, reaching only 63.10 ± 5.76% and 53.53 ± 4.82%, respectively. These results indicated that strain JYK2 degraded lipids more efficiently at lower initial concentrations (≤ 10 ml/l). Fatty acids degradation patterns differed slightly from those of lipids, with continued steady degradation after day 9 even at high initial concentration groups, unlike the plateau observed with lipids during the same period. Degradation ratio in the 5 ml/l and 10 ml/l groups was higher than in the high concentration groups, increasing rapidly during the first 4 days and ultimately reaching 87.45 ± 8.62% and 85.02 ± 10.33%, respectively, by day 12. As higher concentrations (20 ml/l and 30 ml/l), degradation proceeded more slowly, with removal ratio gradually increasing to 68.89 ± 4.46% and 43.78 ± 2.72%, respectively. For glycerol, lower initial glycerol concentrations (1 ml/l) also led to more efficient degradation, with almost completed degradation after day 9 and a final degradation ratio of 93.52 ± 3.97% by day 12. At 2 ml/l, the degradation ratio was slightly lower but still reached 84.24 ± 3.90% by day 12. For higher glycerol concentrations (3–4 ml/l), a lag phase was observed at the beginning of degradation and resulted in lower overall degradation efficiency, with peak value of 65.99 ± 1.85% and 60.24 ± 1.52%, respectively, by day 12.

#### Substrate loss and bacterial growth with lipids and their hydrolysis byproducts

**Fig. 3:**
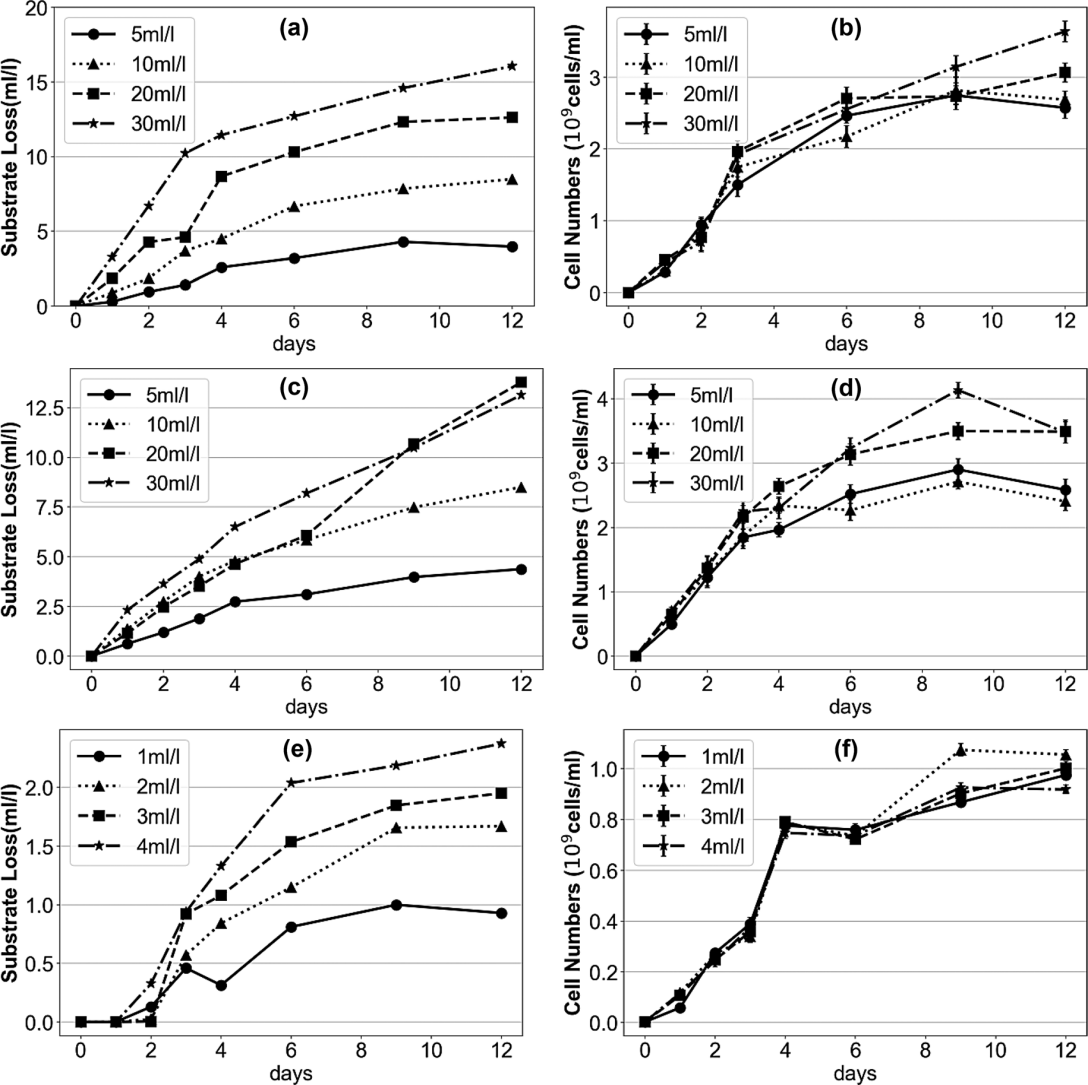
Substrate loss and bacterial growth in different substrates: lipid (**a**) & (**b**); fatty acids (**c**) & (**d**); glycerol (**e**) & (**f**)

Figure 3 revealed the relationship between substrate consumption and bacterial growth in systems containing lipids, fatty acids, and glycerol. A consistent pattern emerged across all substrate systems despite higher substrate consumption at higher initial concentrations, bacterial growth was not proportionally enhanced. In lipids systems (Fig. 3(a) & (b)), higher initial concentrations led to greater substrate consumption, reaching 3.968 ± 0.509, 8.488 ± 0.718, 12.621 ± 1.153, 16.060 ± 1.447 ml/l from lowest to highest concentrations. However, bacterial cell numbers at higher concentrations were only marginally higher than at lower concentrations: 2.575 ± 0.149, 2.688 ± 0.122, 3.067 ± 0.134, and 3.639 ± 0.147 × 10^9^ cells/ml, respectively. Similarly, in fatty acids groups (Fig. 3(c) & (d)), substrate consumption at each initial concentration within 12 days reached 4.372 ± 0.431, 8.502 ± 1.033, 13.777 ± 0.898, 13.134 ± 0.817 ml/l separately, while the cell numbers ranged only from 2.405 ± 0.146 to 3.490 ± 0.176 × 10^9^ cells/ml across the different groups. For glycerol systems (Fig. 3(e) & (f)), substrate consumption over 12 days ranged from 0.929 ± 0.040 to 2.737 ± 0.061 ml/l from lowest to highest concentrations, with cell numbers ranging narrowly from 0.918 ± 0.017 to 1.056 ± 0.020 × 10^9^ cells/ml. These results suggest that as substrate concentration increased, a greater proportion of the energy derived from substrate consumption was directed toward processes other than cell proliferation.

### Production and characterization of EPS by strain JYK2

#### Production of EPS

**Fig. 4:**
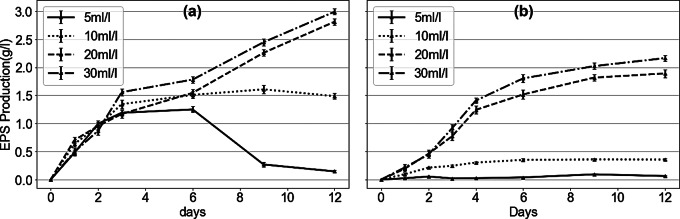
EPS production by JYK2 at different substrates in lipids (**a**) and fatty acids (**b**)

EPS is an important byproduct that influences biodegradation. To study EPS production at different initial concentrations and with different substrates, the mass of EPS in cell-free supernatant was measured periodically (Fig. 4). Among the three substrates tested, glycerol did not support EPS production. Fig. S4 illustrates that EPS levels in all glycerol concentrations remained comparable to the negative control. This may be attributed to glycerol being a small, easily metabolized molecule that can be readily assimilated by bacteria. In lipid-containing media (Fig. 4(a)), EPS was produced at all substrate concentrations. EPS mass increased over time at 20 ml/l and 30 ml/l initial oil concentrations, peaking at 2.813 ± 0.058 and 2.992 ± 0.052 g/l, separately by day 12. However, at 5 ml/l, despite a substantial increase during the first 3 days followed by a stabilization phase from day 3 to day 6, a sharp decline happened thereafter, reaching 0.144 ± 0.028 g/l by day 12. This phenomenon might be due to the utilization of EPS as a carbon and energy source when substrate availability becomes limited. The EPS production pattern at 10 ml/l oil concentrations showed a different trend: rapid initial growth during the first 3 days, followed by fluctuation around 1.486 ± 0.051 g/l for the remaining duration of the study. For fatty acid (Fig. 4(b)), substrate concentration significantly affected EPS production patterns. Minimal EPS was produced at 5 ml/l, and only slightly production (up to 0.356 ± 0.018 g/l by day 12) was observed at 10 ml/l. In contrast, substantial EPS occurred at higher concentrations. At 20 and 30 ml/l, EPS mass increased dramatically during the first 4 days and gradually reached 1.891 ± 0.072 and 2.165 ± 0.051 g/l, respectively, by day12.

#### Composition of EPS

**Fig. 5:**
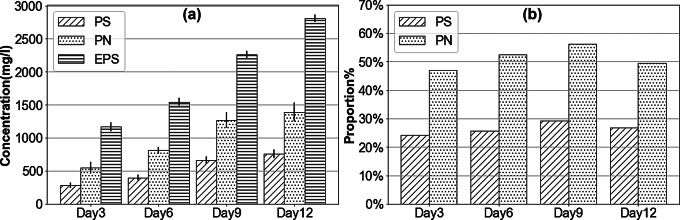
Composition of EPS produced by JYK2. (**a**) and (**b**) refer to the concentrations and proportions of polysaccharides (PS) and proteins (PN) in EPS separately at the initial lipids concentration of 20 ml/l

The composition and function of EPS produced by strain JYK2 were determined in media containing 20 ml/l lipid. The amount and proportion of polysaccharides and protein in EPS were shown in Fig. 5. Figure 5(a) showed the change in mass of polysaccharides, protein, and total EPS over time. All three substances increased throughout the 12-day period, ultimately reaching 755 ± 64, 1390 ± 139 and 2813 ± 58 mg/l, respectively. As shown in Fig. 5(b), the proportion of polysaccharides and protein in EPS did not show a statistically significant difference compared to the overall average, as determined by one-way ANOVA (*n* = 3, *p* > 0.05), with polysaccharides accounting for 24.13 ~ 29.22% and protein for 47.02 ~ 56.27%. These two components, typically considered the main component of EPS, comprised approximately 75% of the total EPS mass in this study.

#### Function of EPS

The functions of EPS produced by strain JYK2 were investigated by analysing its biosurfactant activity and lipase activity. Biosurfactant activity was verified using the oil displacement assay (ODA) method, which is recognized as a fast and reliable analytical method for biosurfactant verification (Saruni et al. 2019). Fig. S4 demonstrated that a clear zone appeared in the EPS-containing cell-free supernatant but not in the control, confirming the presence of biosurfactant activity in the EPS produced by strain JYK2. Lipase activity in EPS was assessed by comparing lipase activity in different treatment groups, distinguished by the presence or absence of EPS. Table 3 shows the results of this analysis. The Triton-X-100 treated sample, which contained depolymerized EPS, showed significant lipase activity that differed significantly from the control group (*n* = 3, *p* < 0.01). In contrast, the control sample, containing only soluble substances, showed negligible lipase activity. These results suggested that lipase produced by strain JYK2 exists primarily in a form adsorbed or captured on EPS rather than in solution, likely due to the high protein content of the EPS, which enhances its hydrophobicity and ability to adsorb lipase molecules.

**Table 3:** Results of the verification experiment on lipase activity in EPS

Group	Contained substances	Lipase activity(U/ml)
Control	Soluble content	1.65 ± 0.07
Triton-X-100	Soluble content + EPS	55.45 ± 2.13

## Discussion

In this study, we isolated various lipid-degrading bacteria, in which *B. arboris* strain JYK2 showed remarkable degradation ability, high lipase activity, and produced substantial amounts of insoluble EPS produced. We conducted a series of experiments to characterize strain JYK2 and observed that hydrophobic substrates significantly influenced EPS production.

Ccompositional analysis demonstrated that polysaccharide and protein accounted for approximately 75% of the total EPS mass produced by *B. arboris* JYK2, with proteins comprising about 50% of the total weight. The proteins-to-polysaccharides ratio (PN/PS ratio) ranged from 1.84 to 2.04 (Fig. S5). These two components typically constitute 60 ~ 90% of EPS (Babiak and Krzemińska 2021; Huang et al. 2022), though their proportions vary under different conditions. Extensive research has demonstrated that hydrophobic substrates promote the production of protein-rich EPS. For example, Corsino et al. (2015) and Wang et al. (2023) obtained protein-rich EPS when sludges were cultured in hydrocarbon-containing slops and lipid-rich wastewater, respectively. Proteins are the dominant hydrophobic components in EPS, whereas polysaccharides are considered as hydrophilic (Geyik et al. 2016; Yang et al. 2023). Higher PN/PS ratios enhance EPS hydrophobicity, surface activity, and aggregate formation (Liao et al. 2001; Xu et al., 2011). The protein in the EPS contributed not only to cell surface attachment but also to biofilm matrix stabilization and development of three-dimensional biofilm architecture (Santschi et al., 2020). The hydrophobicity of protein ultimately enhances the stability of the EPS matrix structure, enabling EPS with high PN/PS ratio to provide protection against adverse environmental conditions (Huang et al. 2022; Sheng et al. 2010). Based on these findings, we hypothesize that the production of protein-rich EPS was attributed to the high concentration of lipids in the culture medium.

As shown in Fig. 4(a), EPS concentration decreased significantly in the later stage of biodegradation in the 5 ml/l of lipids group, possibly due to the nutrient function of EPS. Protein-rich EPS exhibits increased hydrophobicity and adhesiveness, facilitating interactions with other particles or compounds (Santschi et al. 2021). Thus, the hydrophobicity of protein-rich EPS enhances the adsorption of lipids and fatty acids, allowing these immobilized substrates to be utilized by microorganisms under nutrient-limited conditions, resulting in a gradual decrease in EPS mass. Sheng et al. (2010) reviewed that hydrophobic regions of EPS facilitate the adsorption of various hydrophobic organic substances. Besides adsorbed lipids, many researchers have shown that polysaccharides and protein in EPS can serve as nutrient source and be biodegraded by microorganisms. Pirog et al. (1997) mentioned that *Acinetobacter* sp. was able to utilize polysaccharides in EPS during carbon deficiency, and Patel et al. (1974) reported that *Rhizobium* sp. could use extracellular polysaccharides as a carbon source in the absence of other exogenous carbon as well. In addition, Wang et al. (2015) utilized stable isotope probing to label the EPS produced by *Beijerinckia indica* and found that it can be assimilated by the producing strain itself. In this study, bacterial density in low concentration cultures was very similar to that in high concentration cultures during the initial stage, indicating comparable carbon source requirement. However, EPS breakdown likely occurred as bacterial utilized EPS as a secondary nutrient source in substrate-limited environment. We suppose that the decomposition of EPS was attributed to the biodegradation of various components in EPS, including polysaccharides, proteins, and adsorbed lipids.

*B. arboris* strain JYK2 produced lipases in culture systems containing lipids or fatty acids but not in those containing glycerol (Fig. 1). While lipase primarily acts on the ester bond of triacylglycerols to catalyze their hydrolysis into fatty acids and glycerol, fatty acids (the hydrolysis products of triacylglycerol) also induced lipase poroduction, depsite lipase not being directly involved in fatty acid biodegradation. Other researchers have reported similar findings. Shabtai and Daya-Mishne (1992) studied the lipase produced by *Psuedomonas aeruginosa* YS-7 and concluded that both soybean oil and free fatty acids, such as oleic acid and lauric acid, can induce lipase production. Mahler et al. (2000) also confirmed that *Acinetobacter calcoaceticus* could produce lipase in media containing only oleic acid and lactic acid as carbon source. At genetic level, Zeng et al. (2024) figured out that oleic acid can influence the quorum sensing system to control lipase production. By secreting and sensing the signal molecule N-acyl homoserine lactone (AHL) involved in quorum sensing, the lipase-encoding gene is activated, ultimately resulting in lipase production. In contrast, glycerol, a typical hydrophilic substate, did not induce lipase production (Fig. 1). The same phenomena were reported in other studies (Boekema et al., 2007; Shabtai and Daya-Mishne 1992).

**Fig. 6:**
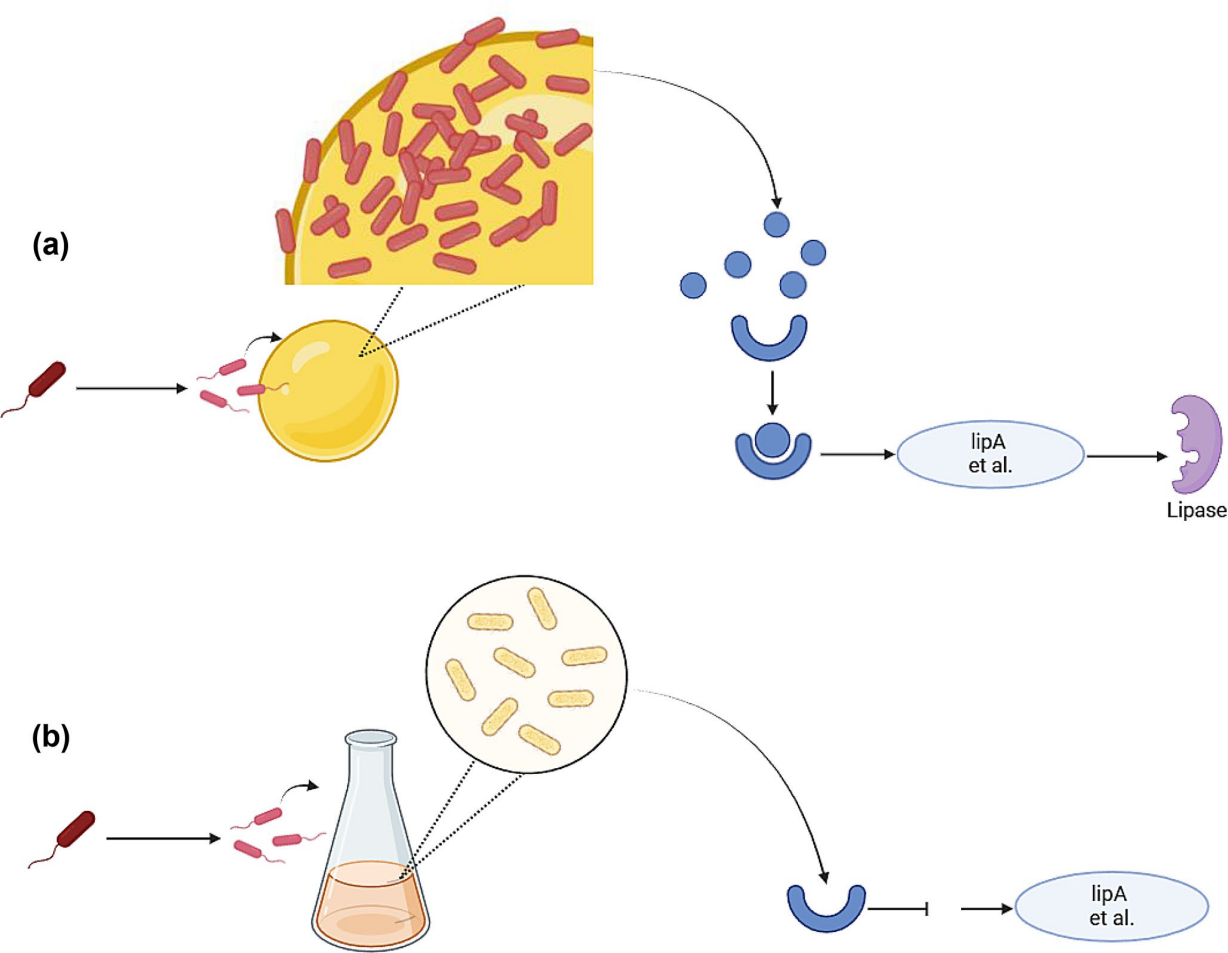
Proposed mechanism of lipase production by *B. arboris* strain JYK2. (**a**) In lipids and fatty acids containing media: (1) Bacteria aggregate around lipid droplets; (2) Local bacteria density increase on oil-water interface; (3) Quorum sensing signal molecules were combined with relevant receptors and later trigger the expression of lipase & EPS production gene such as *lipA*; (4) Lipases are produced. (**b**) In glycerol containing media: (1) Bacteria are evenly distributed in media; (2) Bacteria density is low, and thus, quorum sensing signal molecules do not exceed the threshold concentration; (3) Lipases are not produced

The difference in lipase production observed with hydrophobic versus hydrophilic substrates in the culture medium of strain JYK2 (Fig. 1) is likely due to bacterial condensation on the surface of hydrophobic substrates. Based on previous studies (Devescovi et al. 2007; Rosenau & Jaeger, 2000; Zeng et al. 2024), we propose the following mechanism for lipase production by *B. arboris* strain JYK2 (Fig. 6): at the start of cultivation, lipids and fatty acids form oil droplets on the surface of the liquid medium due to their hydrophobicity. Bacteria aggregate and grow around these droplets due to cell surface hydrophobicity (Dorobantu et al. 2004; Sass et al. 2022; Chakraborty et al. 2010), leading to the increased local bacterial density. and quorum sensing signal molecules concentration. When bacterial density reaches a critical threshold on the oil droplet surfaces, the QS (quorum sensing) system, which regulateds microbial communication based on population density (Miller and Bassler 2001), is activated. The QS system induces the expression of various genes (Zeng et al. 2024), including lipase-encoding genes, through the secretion of signal molecules such as acyl homoserine lactones(AHL) (Fig. 6(a). In contrast, with hydrophilic glycerol, this substrate is uniformly distributed in the liquid medium, preventing localized bacterial aggregation during the 6-day cultivation period. Consequently, signal molecule concentration remains below the threshold required for QS system activation, and no lipase is produced in the glycerol-based system (Fig. 6(b).

EPS was abundantly produced in the presence of hydrophobic substances, such as lipids and high concentration of fatty acids, indicating that substrate type significantly influences EPS production. Compared to hydrophilic substances (Fig. 4 and Fig. S4), EPS production was enhanced in the presence of hydrophobic substances. This phenomenon can be generally attributed to the microorganisms’ response to environmental stress, producing EPS to mitigate toxicity and improve the bioavailability of hydrophobic substrates (Wang et al. 2023). Hydrophobic substances tend to aggregate and form immiscible phases, limiting substrate utilization. Therefore, EPS with biosurfactant activity is produced to facilitate the emulsification and dispersion of these hydrophobic substrates, increase their surface area and bioavailability (Yesankar et al. 2023). In contrast, hydrophilic substances like glucose and glycerol are readily accessible to microbes without requiring such adaptations, reducing the necessity for extensive EPS production. Interestingly, minimal EPS was produced at low initial fatty acid concentrations, suggesting that EPS biosynthesis is not solely regulated by quorum sensing (QS). Kievit (2009) reviewed that among various polysaccharides in EPS, only one subset was QS-regulated, while others were controlled by different mechanisms. We hypothesize that the lower production of EPS at low concentrations of fatty acid might be attributed to differences in the affinity between EPS-producing gene receptor and lipid versus fatty acid. The interaction between fatty acids and their receptors may be weaker than that between lipids and receptors, limiting EPS production at low fatty acid concentrations. However, no relevant research currently exists to verify this hypothesis. Therefore, the mechanism of EPS production by strain JYK2 requires further investigation.

According to the results illustrated in Table 3 the lipase produced by strain JYK2 existed primarily in an immobilized form rather than in solution. This phenomenon may be because protein-rich EPS exhibits higher hydrophobicity and adhesiveness, facilitating interactions with other particles or compounds (Santschi et al. 2021). A substantial fraction of lipases likely interacted with the surface of the EPS and adsorbed to the protein-rich EPS, as evidenced by the high lipase activity detected in the aggregates. This observation aligns with previous studies. Frølund et al. (1995) reported that adsorption immobilized various exoenzymes, including lipase, in sludge EPS. In addition, Yu et al. (2008) studied various extracellular enzymes in sludge EPS and found that most enzymes were detected in the tightly bound EPS layers. This phenomenon may be attributed to the synergistic adsorption capacity of polysaccharides and proteins in EPS. Some enzymatically active proteins associate with the polysaccharides EPS matrix, and this localization helps ensure proximity to substrates and enhance catalytic efficiency (Flemming et al., 2010).

The dynamic changes in EPS concentration suggest a closer link between EPS production and microbial growth. As illustrated in Figs. 3 and 4, EPS production and lipase activity correlated with bacterial cell numbers, consistent with several previous findings (Jia et al. 1996; Sheng et al., 2005; Sheng and Yu et al. 2006a; Sheng et al. 2006b) which demonstrated that lipase, EPS, and other metabolites are produced during the growth phase, with production correlating with cellular growth rate. In contrast, neither bacterial growth nor EPS and lipase production corresponded proportionally to substrate consumption as initial lipid and fatty acid concentration increased, as evidenced by the gradually decreasing overall cell yield with increasing initial concentration (Fig. S6). Ahmed et al. (2022) and Ibrahim et al. (2020) reported similar results. A possible explanation is that microbes require more energy to counteract environmental stressors, leading to increased substrate consumption, as reviewed by various researchers (Jia et al. 2014; Tan et al. 2022; Wang et al. 2024). In our case, elevated long-chain fatty acid concentration may act as an environmental stressor for strain JYK2 due to their cellular toxicity (Salam et al. 2012; Shin et al. 2003).

## Conclusions and future prospect

In this study, we isolated *B. arboris* strain JYK2, a lipid-degrading bacterium capable of producing highly active lipase. Triacylglycerols and fatty acids induced lipase production in this strain. In the presence of these lipid substances, biosurfactant-active EPS was produced, forming emulsion that facilitates the bioavailability of hydrophobic substances. However, the reduced EPS production at low initial fatty acid concentration suggests that the EPS production mechanism cannot be attributed solely to quorum sensing. Compositional analysis revealed that the EPS consisted of approximately 50% protein and 25% polysaccharides. In substrate-limited environments, the EPS produced by strain JYK2 could be utilized as carbon source to maintain metabolic activities. Notably, increasing initial substrate concentration led to higher consumption without corresponding increases in bacterial growth, suggesting that additional energy was expended in high-substrate environments to counter environmental stress. In future studies, we plan to develop mathematical models to quantitatively describe the relationship between bacterial growth and substrate consumption under environmental stress conditions. To verify our hypothesis that bacterial cells aggregate around lipid droplets, we intend to conduct bacterial adhesion to hydrocarbon (BATH) assay to evaluate the cell surface hydrophobicity of *B. arboris* strain JYK2. Furthermore, more sophisticated analytical techniques, such as gas chromatography–mass spectrometry (GC-MS), will be employed to investigate the specific components of EPS, aiming to confirm its enzymatic and biosurfactant activities.

## Electronic supplementary material

Below is the link to the electronic supplementary material.

Supplementary Material 1

## Data Availability

No datasets were generated or analysed during the current study.
