# Supplementary material for: Production of lipase and extracellular polymeric substances by the lipid-degrading bacterium Burkholderia arboris strain JYK2 in response to different substrates
Source: World J Microbiol Biotechnol. 2025 Jun 12;41(6):198. doi: 10.1007/s11274-025-04432-5 (PMC12162804; doi:10.1007/s11274-025-04432-5)

## **Production of lipase and extracellular polymeric substances by the lipid-degrading bacterium *Burkholderia arboris* strain JYK2 in response to different substrates**

Jiyu Huang^1^, Mei-Fang Chien^1^, Hernando P. Bacosa^2^ and Chihiro Inoue^1^,

^1^ Graduate School of Environmental Studies, Tohoku University, Aoba Ku, Aoba 6-6-20 Aramaki, Sendai, Miyagi, 9808579, Japan

^2^Department of Environmental Science, School of Interdisciplinary Studies, Mindanao State University-Iligan Institute of Technology, Andres Bonifacio Avenue, Iligan 9200, Philippines

Supplementary Materials

Fig. S1 Degradation of lipid by the microbial consortia sampling from different locations. K- Kesennuma, F-Fukushima, A-Aobayama, T-Tome, I-Imozawa and Ctrl-Control


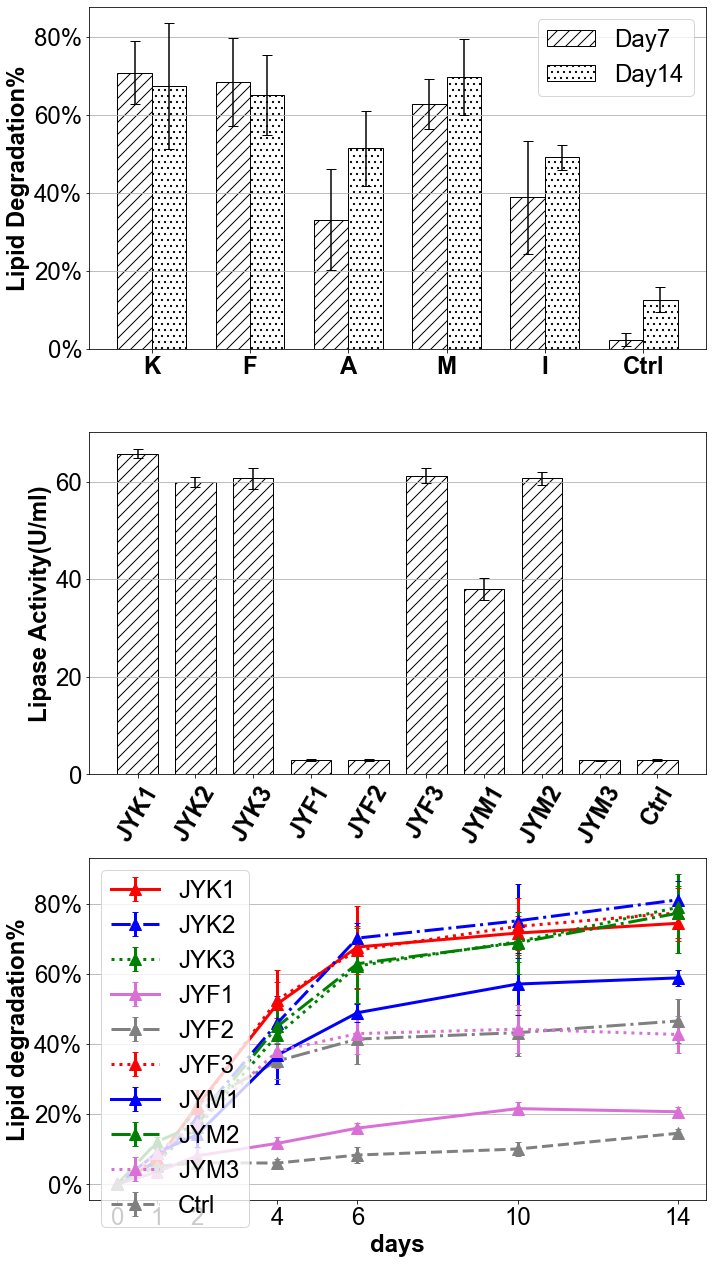


Table S1 Closest relatives in the database of isolated strains and their similarity

| Strain | | Closest Relative | Similarity | Accession of related species |
| --- | --- | --- | --- | --- |
| K | JYK1 | *Burkholderia arboris* strain R-24201 | 99.64% | NR_042634.1 |
|  | JYK2 | *Burkholderia arboris* strain R-24201 | 99.71% | NR_042634.1 |
|  | JYK3 | *Burkholderia arboris* strain R-24201 | 99.86% | NR_042634.1 |
| F | JYF1 | *Methylobacterium radiotolerans* JCM 2831 | 99.93% | NR_074244.1 |
|  | JYF2 | *Klebsiella aerogenes* strain NBRC 13534 | 99.72% | NR_113614.1 |
|  | JYF3 | *Burkholderia arboris* strain R-24201 | 99.71% | NR_042634.1 |
| M | JYM1 | *Serratia marcescens* strain NBRC 102204 | 98.44% | NR_114043.1 |
|  | JYM2 | *Burkholderia arboris* strain R-24201 | 99.86% | NR_042634.1 |
|  | JYM3 | *Klebsiella aerogenes* strain NBRC 13534 | 99.72% | NR_113614.1 |

Fig. S2 Degradation of lipid by the isolated strains over 14 days of incubation.


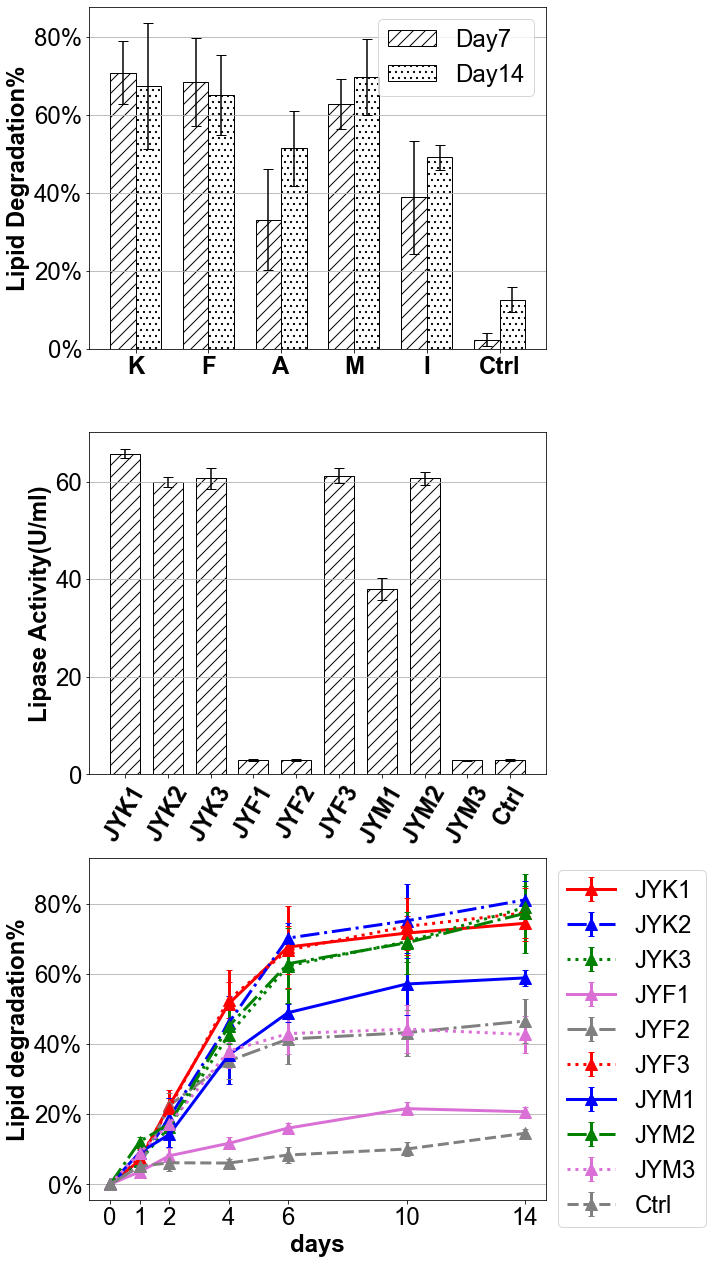


Fig. S3 Degradation ratio of different substrates at different initial concentrations by strain JYK2. (a) referred to lipid, (b) referred to fatty acid and (c) referred to glycerol


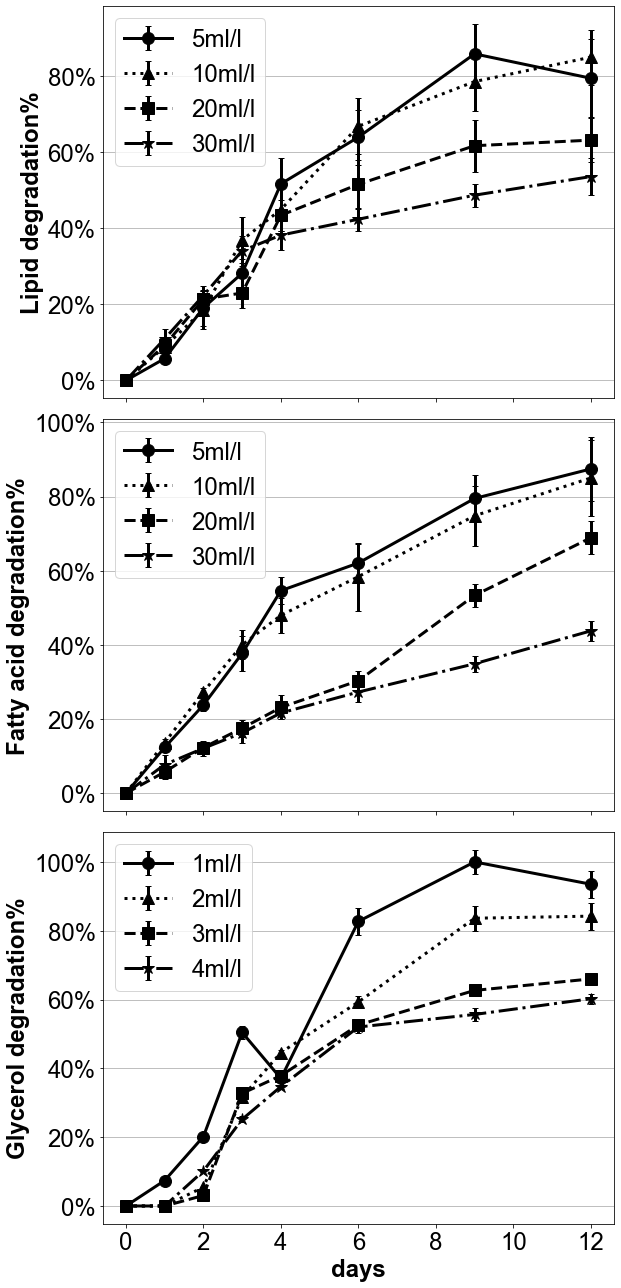


**(a)**

**(b)**

**(c)**

Fig. S4 EPS production in glycerol by strain JYK2


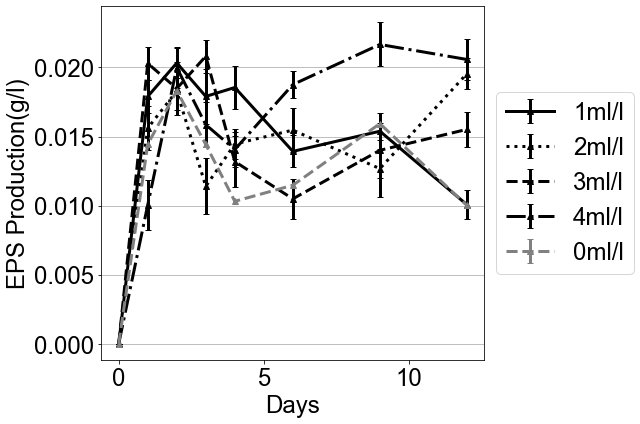


Fig. S4 Result of ODA experiment. The left petri dish was added with cell-free JYK2 suspension and showed an obvious clear zone, while the right one with Milli-Q added but no clear zone was formed.


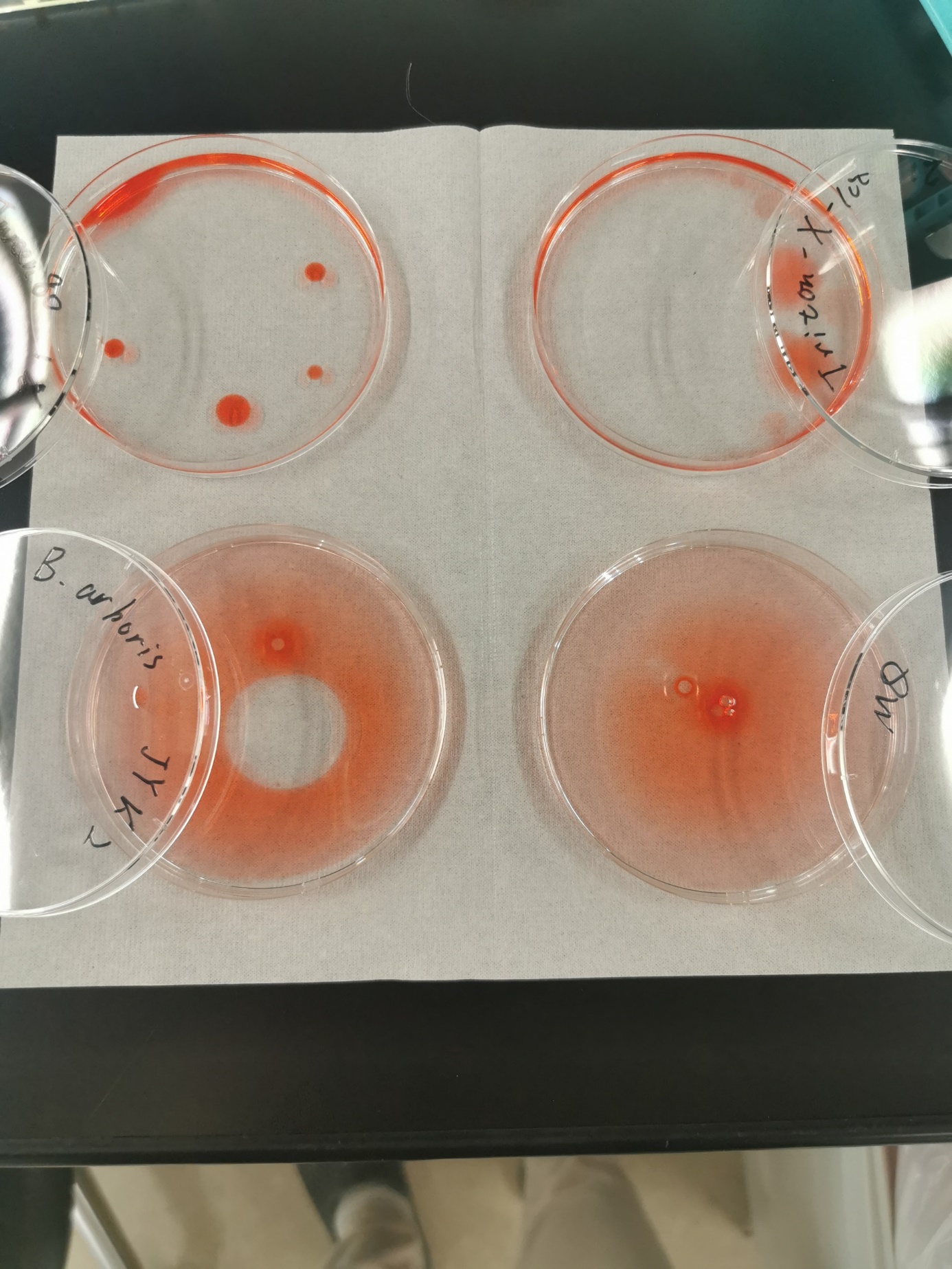


Fig. S5. Ratio of protein to polysaccharide content (PN/PS ratio) in EPS in different periods at 20ml/l of lipid


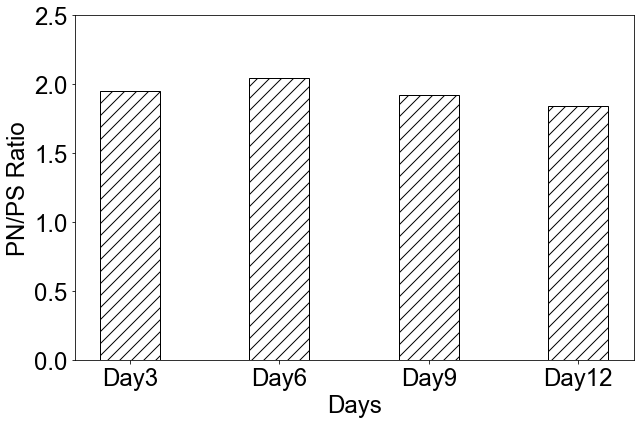


Fig. S6. Overall cell yield within 6 days in different substrates at different substrate concentrations


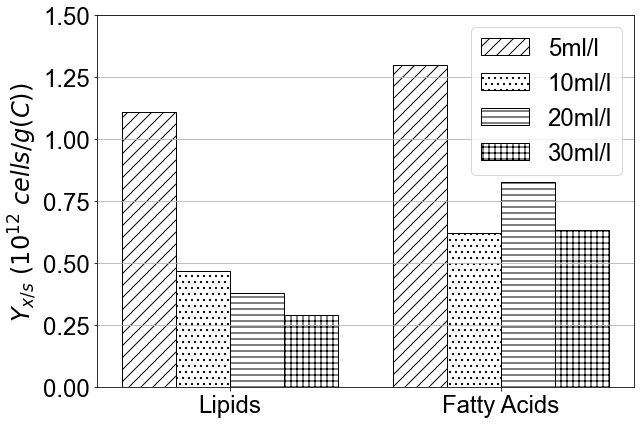

Supplement: Supplementary file 1 — Supplementary Material 1 [file 11274_2025_4432_MOESM1_ESM.docx]
